# Impact of organised programs on colorectal cancer screening

**DOI:** 10.1186/1471-2407-8-104

**Published:** 2008-04-15

**Authors:** François Eisinger, Laurent Cals, Anne Calazel-Benque, Jean-Yves Blay, Yvan Coscas, Sylvie Dolbeault, Moïse Namer, Xavier Pivot, Olivier Rixe, Daniel Serin, Claire Roussel, Jean-François Morère, The EDIFICE committee

**Affiliations:** 1 Paoli Calmettes Institute; INSERM UMR599 232 Bd, St Marguerite, 13009 Marseille, France; 2 Hopital Font-Pre, 1208 avenue du Colonel Picoy, 83000 Toulon, France; 3 Capio Clinique du Parc, 105 rue Achille Viadieu ,31400 Toulouse, France; 4 Hopital Edouard Herriot, 5 Place Arsonval, 69003 Lyon, France; 5 Clinique de la Porte de St Cloud, 30 rue de Paris, 92100 Boulogne, Billancourt, France; 6 Institut Curie, 26 rue d'Ulm, 75005 Paris, France; 7 Centre Azureen de cancérologie, 1 place du Docteur Jean-Luc Broquerie, 06250 Mougins, France; 8 University Hospital Jean Minjoz, Department of Medical Oncology, INSERM U645, 3 Bd Alexandre Fleming, 25000 Besançon, France; 9 Hopital Pitie Salpetriere, 47–83 Bd de l'Hopital, 75651 Paris, Cedex 13, France; 10 Institut St Catherine, 1750 Chemin du Lavarin, 84000 Avignon, France; 11 ROCHE SAS, 52 Bld du Parc, 92521 Neuilly sur Seine Cedex, France; 12 CHU Avicenne, Université Paris XIII, 125 route de stalingrad, 93009 Bobigny, France

## Abstract

**Purpose:**

Colorectal cancer (CRC) screening has been shown to decrease CRC mortality. Organised mass screening programs are being implemented in France. Its perception in the general population and by general practitioners is not well known.

**Methods:**

Two nationwide observational telephone surveys were conducted in early 2005. First among a representative sample of subjects living in France and aged between 50 and 74 years that covered both geographical departments with and without implemented screening services. Second among General Practionners (Gps). Descriptive and multiple logistic regression was carried out.

**Results:**

Twenty-five percent of the persons(N = 1509) reported having undergone at least one CRC screening, 18% of the 600 interviewed GPs reported recommending a screening test for CRC systematically to their patients aged 50–74 years. The odds ratio (OR) of having undergone a screening test using FOBT was 3.91 (95% CI: 2.49–6.16) for those living in organised departments (referent group living in departments without organised screening), almost twice as high as impact educational level (OR = 2.03; 95% CI: 1.19–3.47).

**Conclusion:**

CRC screening is improved in geographical departments where it is organised by health authorities. In France, an organised screening programs decrease inequalities for CRC screening.

## Background

Evidence of the efficacy of screening for colorectal cancer (CRC), in terms of both reduced mortality and reduced incidence through removal of adenomatous polyps, led both the U.S. Preventive Services Task Force [1] and the Advisory Committee on Cancer Prevention in the European Union [2] to recommend mass screening. Colorectal cancer organised screening is increasing at different regional and national levels [3]. In 1998 the French National Consensus Conference on Colorectal Cancer distinguished three levels of risk (moderate, high or very high) and advocated the use of Hemoccult II for mass screening of subjects with moderate risk [4]. Based upon academic initiatives, early studies have been carried out in 3 French departments since 1998 or earlier [5]. Later on, the French national cancer plan focused on screening interventions, including CRC and, from 2003 onwards, regional organised screening programs were set up within a national plan with the objective of nationwide coverage by the end of 2007 [6]. In these programs, biennial faecal occult blood test (FOBT) is first provided by Gps, free on charge, to all subjects aged 50 to 74 years. Over a 4 to 6 month period, the test is mailed to non participants with eventualy a reminder letter. The ongoing progressive implementation of colorectal cancer screening in France affords the unique opportunity to look at differences in compliance, knowledge of population and physicians attitudes between areas with or without organised screening programs.

The EDIFICE nationwide survey was carried out in early 2005 to provide a snapshot of cancer screening procedures in France in 4 selected cancer indications, including CRC. Results of this survey for CRC screening are presented hereunder.

## Methods

### Framework

France administration (including Health administration) is divided into 20 "Regions" (Equivalent to Provinces in Canada or Landers in Germany but with less empowerment than states in the USA) and 95 "Departments". The mean number of inhabitants is 3,1 million for Regions and 650 000 for Departments.

When organised, disease screening is currently carried out at the departmental level after decision at the national level. Once a decision is made about which services to offer and to whom (decision and funding at the national level), the local health administration submit to the Health Ministry an authorization to start the program, once fulfil all the specifications described by the Ministry. For colorectal cancer the specifications mainly are the following: Training of GP, an information letter to every affiliated to the National Health Insurance System (almost every person living in France) age 50–74y, no more than one center to analyse FOBT by department, the utilization of Hemoccult, description of criteria for not undergone FOBT (among which familial history of Colorectal cancer...).

Therefore there is a national way to organised screening, but local differences about when the program started.

### General Population survey

A nationwide observational survey (opinion poll) was carried out by telephone from January 18^t ^to February 2, 2005 among a representative sample of subjects living in France and aged 40–74 years. Representativeness of the survey sample for gender, age, profession and double stratification by geographical area and community size as compared to the French general population was ensured by the use of the method of quotas [7], based on the statistics of the French Employment Survey conducted in 2002 by the French National Institute for Statistics and Economical Studies (INSEE). The 170-item survey questionnaire was administered by trained and independent interviewers of TNS-Healthcare SOFRES using the Computer-Assisted Telephone Interview (CATI) technique. Telephone interviews lasted 25 minutes on average. On account of the size of the questionnaire, questions concerning four cancers studied (breast, colorectal, prostate and lung cancer) were rotated during the successive telephone interviews. The survey questionnaire collected information about subjects' socio-demographic characteristics (gender, age, residence area, community size), attitude and behaviour regarding cancer screening (in general and for the four organs concerned), actual experience of cancer screening, and attitude as regards personal health (self-medication, perceptions on vaccination, medical consultation during the past year, tobacco and alcohol consumption). The questionnaire distinguished tests performed for screening purpose and those performed following symptoms. A main sample of 1 509 subjects aged between 40 and 74 years was interviewed. An additional sample of 100 subjects aged between 50 and 74 years (recommended age interval for the screening of CRC) was also interviewed in order to obtain a representative number of subjects living in the 22 French departments involved in organised CRC screening programs. Computerised weighting [8] of the whole sample of 1 609 subjects allowed for compensation of under-representation of the additional sample in the whole sample (adjustment to the proportion of all subjects living in the 22 departments involved in organised CRC screening programs). Subjects with a personal history of cancer (N = 105) were excluded from analysis because actual experience of cancer might affect cancer screening perceptions. Therefore, the whole subject sample analysed was comprised of 1 504 individuals aged between 40 and 74 years, among whom 970 subjects of both genders aged 50–74 years were interviewed for CRC screening. Precision of results for this sample was ± 3.2% with 95% confidence interval (CI).

### Survey among General Practitioners

The survey (opinion poll) was carried out by telephone from January 31 to February 18, 2005 among a representative sample of French general practitioners (GPs). Representativeness of the survey sample for age and region of residence (five regions) as compared to the national population of GPs was ensured by the use of the method of quotas [7]. The 45-item survey questionnaire collected information about GPs' socio-demographic characteristics (gender, age, department of France) and their medical practice regarding screening of cancer (breast, colorectal, prostate, and lung cancer), especially perceptions on screening methods, level of screening counselling, screening tests recommended, perceived obstacles to screening, and persons'expectations about cancer screening according to GPs. Six hundred GPs were interviewed in order to obtain a sufficient number of GPs practicing in the departments of France involved in planned screening of colorectal cancer (N = 178; 30%).

### Statistical analysis

The departments were divided into two categories according to the existence or absence of an organised colorectal cancer screening program. Among the "organised" departments (N = 22), two groups were defined according to the timing of the initial program implementation:

- more than 18 months ago: twelve "first-wave" departments (Côte-d'Or, Ille-et-Vilaine, Saône-et-Loire Charente, Indre-et-Loire, Calvados, Haut-Rhin, Hérault, Isère, Seine Saint Denis, Bouches-du-Rhône, Nord) which started in 2003 or earlier;

- about 12 months ago: ten "second wave" departments (Allier, Ardennes, Essonne, Finistère, Marne, Mayenne, Moselle, Orne, Puy-de-Dôme, Pyrénées-Orientales) which started in 2004.

Data analysis was essentially descriptive. Quantitative data were described by the means and standard deviations (SD) and categorical data by the numbers in each category and corresponding percentages. Statistical comparisons were carried out by the Student's *t *test for quantitative data, and by the Z test and the Chi-square test for the comparison of percentages and numbers, respectively, in the case of categorical data. Differences were considered statistically significant when the probability value was less than 0.05 (bilateral test). Multivariate logistic regression analyses were expressed in terms of odd ratio (OR) and 95% CI and performed using the SAS^® ^software, version 8.2 (proc FREQ and proc LOGISTIC procedures).

## Results

At the time of initiation of the EDIFICE Survey, organised screening programs proposed FOBT in 22 of 95 metropolitan departments, corresponding to an estimated 18,230,000 inhabitants in 2003 or 30% of the national population (or 4,650,000 subjects aged 50–74 years, corresponding also to 30% of the national population in the same age range).

### Subjects' characteristics

The median age of the 970 interviewed subjects was 61 years, 52% were female, 42% lived in towns with > 100,000 inhabitants, 28% lived alone and 89% had visited a physician within the last 12 months.

### Screening tests (Table 1, 2 and 3)

**Table 1:** Declaration of having undergone at least one colorectal cancer screening test according to subjects characteristics.

		**% of subjects declaring screening test**	**P value**
**ALL**	**(n = 970)**	**25%**	

**Gender**			
• Male	(n = 462)	24%	
• Female	(n = 507)	26%	0.47
**Age **(years)			
• 50–54	(n = 213)	19%	
• 55–59	(n = 241)	30%	
• 60–64	(n = 180)	28%	
• 65–69	(n = 179)	27%	
• 70–74	(n = 157)	18%	0.01
**French department of residence**			
• With organised screening	(n = 329)	34%^1^	
• Without organised screening	(n = 641)	20%	<0.01
**Age of organised program**			
• More than 18 months ago ("first-wave")	(n = 220)	37%^2^	
• About 12 months ago ("second-wave")	(n = 109)	26%^3^	0.03

^1^including 17% of screening tests performed within organised programs

^2 ^including^2^21%; ^4^10%, respectively, of screening tests performed within organised programs (p = 0.04)

**Table 2:** Variables increasing the probability of being screened. Univariate analyses

	**Model 1**		**Model 2**	
	Regardless of screening procedure^£^	Not screened	With FOBT + endoscopy	Not screened

	**N = 132**	**N = 838**	**N = 112**	**N = 730**

**Region:**				
Paris and around	11%*	18%	8%*	19%
West	30%	23%	34%*	23%
South West	5%**	12%	6%**	13%
				
**Size of city :**				
Paris or suburb	9%*	16%	7%**	17%
				
**Age :**				
Being 50–54 year old	18%	22%	12%**	24%
Being 55–59 year old	32%	24%	33%*	23%
Being 70–74 year old	11%*	17%	12%	18%
				
**Visited Physicians :**				
Having visited a doctor within the last 12 years	95%**	88%	95%**	88%
Having visited a g-e within the last 12 years	22%**	5%	8%	5%
				
**History of cancer :**				
Cancer(s) in family or close circle	79%**	66%	77%*	66%
Colorectal Cancer(s) in family or close circle	14%*	7%	8%	5%
				
**Other general believes :**				
I make decisions easily	66%*	55%	63%	55%
Concerning my health, I have to face up to my responsibilities	89%*	82%	88%	83%
				
**Opinions/fears about cancer :**				
I think having more cancer risks than most of the people	28%	24%	17%*	24%
Being afraid of colorectal cancer	64%*	51%	60%*	49%
Being afraid of screening tests	22%**	39%	24%**	41%
Giving the adequate definition of screening	63%	55%	65%*	55%
Being confident in screening efficacy	83%**	65%	80%**	63%
Quoting FOBT as a screening test	44%**	15%	57%**	14%
Quoting endoscopy as a screening test	67%**	46%	47%	43%
				
**Opinion about colorectal cancer :**				
Being motivated by CRC screening	74%**	33%	64%**	28%
Being concerned by CRC screening	77%**	39%	72%**	33%
CRC is an important process	83%**	54%	81%**	50%
				
**In the future :**				
Intent to do a screening test in the future	58%*	46%	74%**	43%
-To do so in the "organised screening"	14%	9%	20%**	9%
-Do not care	34%*	21%	42%**	20%
				
**French department of residence :**				
With organised colorectal cancer screening	59%**	30%	64%**	30%

£: either FOBT or endoscopy **p < 0.01 *p < 0.05

List of variables included in the models: Gender, Age, Work status, City of living's size, Educational level, Marital status. Self-medication, Vaccination. Anxiety about his/her health. Screening of breast cancer: screnned (follow-up or not)/never screened. Having visited a GP, a gastroenterologist within the last 12 years. Attitudes towards his/her health (responsibility, take care without delay, no influence, doctor's business). History of cancer (in general and colorectal) in his/her family/close circle. Being afraid by cancer, colorectal cancer, screening tests. Being confident in screening efficacy. Being concerned, motivated by CRC screening, CRC is an important process. Year of instauration of breast cancer screening programThe existence of an organised colorectal cancer screening program

**Table 3:** Variables increasing the probability of being screened. Multivariate analyses

**Variable**	**Odd ratio (95% CI)**
**Regardless of screening procedure (either FOBT or endoscopy)**	

Having visited a gastroenterologist within the last 12 years	5.55 (3.02–10.19)
Living in the 22 departments with organised screening programs	3.89 (2.52–5.98)
Being concerned by CRC screening	2.60 (1.43–4.71)
Being motivated by CRC screening	2.26 (1.27–4.02)
Being confident in screening efficacy	1.98 (1.15–3.40)
Having high educational level (College or higher)	1.74 (1.05–2.90)
Being afraid by results of screening tests	0.47 (0.29–0.77)
Living in Paris or suburb	0.37 (0.15–0.92)

**With FOBT ± endoscopy**	

Living in the 22 departments with organised screening programs	3.91 (2.49–6.16)
Being concerned by CRC screening	3.17 (1.75–5.72)
Having high educational level (College or higher)	2.03 (1.19–3.47)
Being motivated by CRC screening	2.02 (1.13–3.62)
Being afraid by results of screening tests	0.56 (0.34–0.93)
Being 50–54 year old	0.40 (0.19–0.86)

Two hundred and forty subjects (25%) reported having undergone at least one screening test for CRC. Among them, 76% declared having undergone the test based on individual initiative compared to 24% within an organised screening program. The majority (53%) declared having undergone endoscopy alone (without distinction available between colonoscopy and sigmoidoscopy), while 46% report having undergone FOBT ± endoscopy. This trend was reversed in the 22 pilot departments (FOBT 65%; endoscopy alone 35%). Subjects in extreme age categories (50–54 and 70–74 years) declared having undergone significantly less screening tests than other categories (Table 1). Subjects living in the 22 departments with organised screening programs reported having undergone significantly more screening tests than others (OR = 1.99; 95% CI: 1.47–2.69; p < 0.01), including 52% of them within screening programs (Table 1). In these departments, the percentage of subjects declaring having undergone a screening test significantly increased with the age of the local program, from 26% in the most recently implanted, to 37% in the first-wave departments (p = 0.03, OR = 1.76 – IC 95% 1.06–2.93).

### Factors influencing screening test performance

Characteristics of screened and unscreened subjects were compared. In the univariate analysis (Table 2), significantly more unscreened subjects lived alone and lived outside the 22 departments with organised screening. Significantly fewer of them had visited a gastroenterologist within the past 12 months, were concerned/motivated by screening, were afraid of CRC and had cancer or CRC cases among their relatives or friends. Lastly, unscreened subjects had significantly lower incomes than screened subjects. After multivariate logistic regression analysis, eight independent variables influenced screening (six positively and two negatively), irrespective of the test used (FOBT and/or endoscopy) (Table 3). The strongest predictive variable (OR: 5.55; 95% CI: 3.02–10.19) was to have visited a gastroenterologist within the last 12 months. However, when only screening with FOBT is taken into account, only four positive variables remained correlated with screening. Living in one of the 22 departments with organised screening programs was the strongest predictive variable, followed by motivation/concern for CRC screening and educational level, while the influence of gastroenterologists disappeared (Table 3).

Among the subjects declaring having been screened in the 22 pilot departments, 52% did so within an organised program and 46% based upon individual initiative. Almost all subjects (93%) who participated in mass screening programs underwent FOBT, compared to 34% of subjects screened based upon individual initiative (p < 0.01), whereas only 26% underwent endoscopy, compared to 74% (p < 0.01), respectively. Sixty-four percent of subjects who participated in mass screening programs were invited to do so through a mailing campaign from the French Health Care System ("Social Security"). Subjects in screening programs were significantly older at the time of their first screening (57.8 versus 52.3 years; p < 0.01).

### Perception of CRC screening by population

Fifty-six percent of interviewed subjects gave an adequate definition of cancer screening and 88% knew that screening increases the likelihood of CRC cure. In the 22 pilot departments, 86% of interviewed subjects felt the invitation by mail was motivating and only 6% found it worrying. Individuals who did not undergo screening tests were invited to state the reason from a limited pre-established list. Few differences appeared between the two categories of departments. "Feeling of not being concerned" was lower, although not significantly, in organised departments (33% versus 38% of subjects; OR = 0.83; 95% CI: 0.59–1.16);"having no symptoms" was also not significantly lower in organised departments (17% versus 21%; OR = 0.76; 95% CI: 0.50–1.14) and "fear of the test and/or its results" was higher in organised departments (11% versus 6%; OR = 1.97; 95% CI: 1.11–3.49).

### Screening attitudes and perceptions of GPs

Eighteen percent of the 600 interviewed GPs reported recommending a screening test for CRC systematically to their patients aged 50–74 years, while others declared to "often" (48%), "seldom" (28%), or "never" (6%) recommend doing so. The proportion of GPs who reported systematically recommending a test was higher in the 22 pilot departments than in other departments (29% versus 13%; p < 0.01) and increased, but not significantly, with the age of the local program (26% in the "second-wave" departments, and 30% in the first wave depârtments; p = NS, OR = 1.20 – IC95% 0.58–2.51). The main reasons given by GPs for not systematically recommending screening tests (Table 4) were the belief that screening should be restricted to subjects at risk (28%) and the feeling that they were not associated to the general program (19%). On the contrary, GPs considered that patients' reluctance to perform screening tests is related to fear of results (16%), feeling of not being concerned (11%), non-recommendation by GP (9%) or lack of information (8%).

**Table 4:** Reasons given by the Gps for not systematically give the recommendation for screening. N = 492

**Reasons**	**Rate**
Recommendation to âtients at risk only	28%
Not enough involved in the process by official institutions	19%
The ration costs/benefit is high	9%
The difference between costs & benefit is low	9%
Neglect	8%
The National Health Insurance is in charge of this recommendation	5%
Patient's choice	4%
Difficulty of the realisation – The screening tests are uneasy	4%
Screening tests are not very efficient	3%
It is gastroenterologist's role	<1%
Other reasons (others priorities...)	4%

## Discussion

The EDIFICE nationwide survey was carried out to provide a snapshot of cancer screening procedures in France in 4 selected cancer indications, including CRC. This survey relies on self-reported data and does not report the actual incidence of screening tests for CRC. Though the questionnaire was discriminative for true screening, it is likely that some of the reported "screening tests" were actually diagnostic tests following discrete symptoms, especially for tests performed based on physician's prescription. Self-report accuracy of screening tests may be test-dependent and FOBT has been shown to be under-reported [9] but also over-reported [10]. Nevertheless, self-reported screening behaviour is generally fairly accurate [11,12], and many publications rely upon this. However, this survey does have limitations inherent to the design (cross-sectional) or a limited generalizability due to the economic and organisational French background.

The first observation of this survey is the low rate (25%) of reported screening for CRC in France in the target population aged 50–74 years, in contrast with a high level of scientific evidence and official recommendation [1,2]. This low rate is close to [13,14] rates observed in other Western countries, but significantly lower than the figures in recent publications. For instance in 2004, the rate for US adults above 50 y who reproted receiving either a FOBT within one year or an endoscopic examination within 10 years is 57.1% [15].

In contrast to other developed countries [13], the financing of screening tests, whether they are performed individually or within an organised program, is not an issue, since they are all paid for by the French Health Care System.

The second point is the influence of locally organised screening programs on screening attitudes of both population and GPs. The rate of subjects reporting having been screened (either individually or through screening programs), the rate of GPs systematically recommending CRC screening, the proportion of subjects screened undergoing FOBT, compared to endoscopy, as well as that of subjects having performed a test within the last two years, were all increased in departments where an organised screening program exists. Organised cancer screening is indeed assumed to be more effective than opportunistic screening [16]. Furthermore, it has been shown to improve guideline compliance, especially with regard to the adequacy of examinations that should follow positive FOBT [16-18], and therefore are likely to protect subjects from the risk of poor-quality screening practices and to guarantee screening cost-effectiveness [16].

The most important finding of our survey is that the existence of an organised local screening program is the strongest independent predictive factor of performing a screening test in the logistic regression analysis (Table 3). When only FOBT is considered, almost 4 times subjects living in the 22 pilot departments reported undergoing screening tests than those living in other departments and it is anticipated that this difference will grow over time with program implantation. Furthermore, living in a pilot department is almost twice as predictive as educational level (Table 3). This finding suggests that the implementation of organised screening programs minimizes inequality for CRC screening. When either FOBT or endoscopy are considered, the strongest predictive factor is having consulted a gastroenterologist within the last 12 months. This should be put into perspective with the role of endoscopy in individually-based screening procedures. The fact that having consulted a gastroenterologist is no longer an associated factor when considering FOBT and endoscopy minimize the risk of transposition of cause and effect (visits prompted by FOBT).

Within departments where organised programs are implemented the declaration rate of screening tests (37 versus 26%), the reported rate of participation in the local program (21 versus 10%) and the reported systematic recommendation by GPs of performing screening tests (30 versus 26%) were higher in departments in which the local program was first implanted than in those in which it was set up recently. These correlations are likely to be explained by the educational role of organised programs and solicitation of population and physicians. The fact that a decreasing rate of subjects wrongly assume that having no symptoms is a reason for not performing a screening test, supports this assumption. In a US survey, "lack of awareness" and "not recommended by a doctor" were the most common barriers to CRC screening and similarly are decreasing with time [19]. Moreover, the participation rate in screening in the "scout" departments is close to the objective of 50% participation as set in the French Cancer Plan established by the French Health Ministry, and may be the maximum achievable rate with such programs. Nevertheless, these "scout" departments, in which mass screening was initiated based on academic initiative, may be more highly implicated in screening and cannot necessarily be extrapolated to other departments according to national directives.

The role of GPs is important for individual screening practices [19] particurlarly for long term compliance [20]. In France in 2005, only 18% of them systematically recommended CRC screening tests and an additional 48% "often" recommended them. This compares with 59% of GPs recommending tests in a Canadian survey [21]. Surprisingly, the level of knowledge about CRC screening of the general population and GPs seems correlated and similarly influenced by organised local programs. Other yet undetermined disease- or test-related factors may negatively influence CRC screening. It has been shown, for instance, that in a cohort of well informed women, fewer undergo FOBT than mammography for cancer screening [22].

## Conclusion

The rate of CRC screening testing is still low in France, but is expected to increase regularly with the nationwide implementation of mass screening programs, which are likely to be the main factor influencing subjects to undergo, and GPs to systematically recommend, screening tests. Nevertheless, the rate of screening test performance in the areas with the oldest organised programs (> 6 years), about 50%, could be the highest rate achievable with time using this kind of organisation. This could still be considered as insufficient. Further public health research is warranted to clarify remaining barriers and improve the methods of informing the population and GPs [23].

## List of abbreviations

CRC: Colorectal Cancer. GP: general practionner. OR: Odd Ratio. FOBT: faecal occult blood test. CI: confidence interval

## Competing interests

The survey was funded by a grant of ROCHE, Neuilly-sur-Seine, France, and the member of the Edifice committee (all the co-authors of this contribution) received honorarium from Roche.

## Authors' contributions

FE contributed to the design of the survey, to the data analysis, attented all working cession and drafted the manuscript. LC contributed to the design of the survey, to the data analysis, attented all working cession and reviewed the manuscript. ACB contributed to the data analysis, attented all working cession and reviewed the manuscript. JYB contributed to the design of the survey, to the data analysis, attented almost all working cession and reviewed the manuscript. YC contributed to the design of the survey, to the data analysis, attented all working cession and reviewed the manuscript. SD contributed to the design of the survey, to the data analysis, attented all working cession and reviewed the manuscript. MN contributed to the design of the survey, to the data analysis, attented all working cession and reviewed the manuscript. XP contributed to the design of the survey, to the data analysis, attented all working cession and reviewed the manuscript. OR contributed to the design of the survey, to the data analysis, attented almost all working cession and reviewed the manuscript. DS contributed to the design of the survey, to the data analysis, attented almost all working cession and reviewed the manuscript. CR contributed to the design of the survey, carried out the coordination of the team, attented all working cession and reviewed the manuscript. JFM contributed to the design of the survey, to the data analysis, attented all working cession and reviewed the manuscript. All authors read and approved the final manuscript.

## Pre-publication history

The pre-publication history for this paper can be accessed here:


